# A qualitative exploration of the views of paramedics regarding the identification of cardiovascular risk factors in the pre-hospital environment

**DOI:** 10.29045/14784726.2022.06.7.1.19

**Published:** 2022-06-01

**Authors:** Josie Taylor, Graham McClelland

**Affiliations:** North East Ambulance Service NHS Foundation Trust; North East Ambulance Service NHS Foundation Trust ORCID iD: https://orcid.org/0000-0002-4502-5821

**Keywords:** cardiovascular disease, paramedic, risk factors

## Abstract

**Background:**

Cardiovascular disease remains the most prominent cause of death in England. Healthcare professionals have been encouraged to identify cardiovascular risk factors (CVRFs). The aim of this study was to explore how paramedics contribute to the identification of CVRFs in the pre-hospital setting, through their role, behaviours and practice.

**Methods:**

The study took place within the North East Ambulance Service NHS Foundation Trust supported by a National Institute for Health Research clinical research internship. A qualitative approach was used, and a single focus group was conducted. The study recruited participants through advertising for volunteers and purposive sampling. The themes that arose from the focus group allowed the initial exploration of the views of paramedics in relation to role, behaviour and practice in identifying CVRFs.

**Results:**

A single focus group with five paramedics was conducted in June 2021. Two central themes emerged: education/health promotion and fear/anxiety. Participants agreed that their role in this area centred around patient education. Participants’ behaviours and practice were adversely affected through fear of complaints, fear of hypocrisy and feeling a lack of support from the ambulance service. Participants felt that further training and subsequent indemnity from complaints would improve the likelihood of more direct patient education. Support from the ambulance service to improve employees’ own health and well-being was also a key topic of discussion.

**Conclusion:**

The study explored the views of a small sample of paramedics on this topic. Patient education was felt to be part of a paramedic’s role; however, barriers were identified that prevent paramedics from carrying out this role. Further research is needed to explore these barriers further.

## Background

NHS England (2018) defines cardiovascular disease (CVD) as an umbrella term for disease affecting the heart and/or blood vessels. CVD remains the most prevalent cause of death in England (British Heart Foundation [BHF], 2021). Although the mortality rates of CVD are decreasing, these conditions still account for 27% of all deaths in the United Kingdom (BHF, 2021).

The NHS long-term plan (NHS England, 2019b) recognised the gravity of CVD and ensured the development of care in CVD remains a priority: ‘This is the single biggest area where the NHS can save lives over the next 10 years’ (NHS England, 2019b, p. 62).

The NHS has encouraged all staff to take a proactive role in health promotion, with approaches such as ‘making every contact count’ (Health Education England, 2021). While the need for health promotion is clearly demonstrated in the governmental strategies for the NHS (NHS England, 2019a), it is not yet fully understood how paramedics fit into this role.

It is clear from the discussed guidance that paramedics have a role to play in addressing cardiovascular risk factors (CVRFs), but there is currently limited research on this topic. The primary aim of this study was to explore how paramedics contribute to the identification of CVRFs in the pre-hospital setting, through their role, behaviours and practice, with a secondary aim to identify areas paramedics feel can be improved to support their identification of CVRFs.

## Methods

A generic qualitative approach was used to explore the topic in question (Kahlke, 2014). This study was conducted as part of the National Institute for Health Research clinical research internship, thus time constraints needed to be abided by due to the internship lasting 9 months with limited time allocated to carry out the study.

### Participants and setting

Due to the constraints that the COVID-19 pandemic posed to face-to-face data collection, the focus group was conducted virtually via Microsoft Teams^TM^. Participants were recruited from North East Ambulance Service NHS Foundation Trust employees who were registered paramedics. Paramedics were chosen for this project due to the clinical decision-making expertise that this group of clinicians possess. An open advert for voluntary participation was published using the Trust’s intranet which is available to all staff members.

Adverts aimed to recruit 4–6 participants for a single focus group (Kitzinger, 1995). Participants were offered a £10 voucher as compensation for their contribution.

### Data collection

Focus groups were chosen as the method of data collection. Leung and Savithiri (2009) commend the use of focus groups to encourage natural discussion among participants and allow for shared opinions.

A topic guide was devised prior to the focus group (Supplementary 1), to provide a framework for data collection, while allowing free-flow for conversation in keeping with the study aims (McCracken, 1988).

The focus group was digitally recorded to aid the transcription process. Field notes were taken during the focus group to further aid analysis and the transcription process. The main author (JT) conducted the focus group, with GM also present to assist in facilitation of the focus group.

### Data analysis

Thematic analysis was used to analyse the data. Kiger and Varpio (2020) illustrate the stepwise approach that was adopted for this study. The lead researcher (JT) transcribed the data from the focus group verbatim and carried out subsequent analysis. All participant input was anonymised during the transcription process. The lead researcher (JT) is a practising paramedic who was known to the participants. Recognising the potential for personal bias to influence the coding and analysis, efforts were made to reduce this through discussion of the main themes with the supervisor of the project (GM).

## Results

A single online focus group was conducted in June 2021 with five paramedic participants who volunteered following the recruitment advertisement. The main themes identified were the paramedics’ role in patient education, and anxiety in addressing these topics with patients. Participant anxiety centred around the fear of complaints from patients, as well as lack of support from the ambulance service. The focus group consisted of paramedics across the gender spectrum, who had all been qualified between 4 and 10 years. All had experience of working in both urban and rural environments.

### Role

All participants agreed that their role in the identification of CVRFs centred around patient education (Table 1, quotes 1.1 and 1.2). The areas that were heavily discussed were: obesity, lifestyle choices and exercise, summarised as ‘health promotion’.

**Table 1.** Participants’ comments surrounding ‘role’ of the paramedic.

Quotation reference	Quote
1.1	*‘every patient we see, we should be educating’* (participant 2)
1.2	*‘We’re there because of something like angina, we’re there for the bad side, so that’s the right setting’* (participant 5)
1.3	*‘they’ve potentially had an entire upbringing where that’s what they’re used to. It’s like a mindset – you’re not just changing a temporary thing; it’s literally how they’ve grown up’* (participant 5)

Although participants agreed that their role centred around educating patients on health improvement to minimise their CVRFs, there was negligible discussion of more preventative strategies. Further discussion about generational barriers in education led to dialogue surrounding socio-economic status and health inequality (Table 1, quote 1.3).

### Behaviour

Behaviours were affected by three areas: fear of complaints, feeling a lack of support from the ambulance service and fear of hypocrisy (Table 2, quotes 2.1 and 2.3).

**Table 2.** Participants’ comments surrounding ‘behaviour’ of paramedics.

Quotation reference	Quote
2.1	*‘we see them maybe once or twice in a year, for the same issue, but for an hour, and it’s a long term adherence problem’ . . . ‘you turning up for an hour and saying ‘stop eating, stop drinking’, ok yeah whatever. It’s not a switch, it doesn’t flip’* (participant 4)
2.2	*‘I find it easier to tell somebody that their relative has died, than that they’re fat’* (participant 3)
2.3	*‘there should be a debate within the ambulance service of how far we’re going to go, and how strongly we’re going to deliver that message’* (participant 3)
2.4	*‘as long as you’ve completed that training, if the service were to say, “right, we’ll back you, as long as you’ve used our X, Y and Z method of communicating with a patient, we will back you so if that patient gets offended or raises a complaint saying X called me fat, well yes, X did say you were fat, because you are fat and you need to do something about that”’* (participant 1)
2.5	*‘as an ambulance service, we should be role models for society’* (participant 2)
2.6	*‘that might be something that we could get to peak physical fitness, so that we can display ourselves to patients as a pinnacle of physical exercise’ … ‘it would be much easier to broach the subject’* (participant 1)
2.7	*‘it’s hard because CV risk factors aren’t as cut and dry as things like smoking/alcohol referring’* (participant 5)

Fear of complaints affected the participants’ behaviours in practice (Table 2, quote 2.4). Although participants agreed feeling confident in broaching subjects such as smoking cessation or alcohol reduction with patients, the taboo subject almost unanimously agreed among participants was addressing patients’ weight (Table 2, quotes 2.2 and 2.7).

The participants felt they risked receiving complaints and felt the ambulance service would not support their actions, thus reducing the chances of the participants broaching the subjects with patients. Participants suggested an area for development to include training courses that include how to broach contentious subjects, such as weight, with patients (Table 2, quotes 2.2 and 2.4).

Another widely discussed subject was feeling hypocritical when discussing health promotion with patients. They stated that they would feel uncomfortable discussing a patient’s weight, if they, or a colleague that was present with them, were overweight themselves (Table 2, quote 2.5).

One participant felt that support could be offered to staff by means of fitness facilities and time on shift to use said facilities. This was said with a clear sense of humour, as participants felt this was an unobtainable solution due to service demand (Table 2, quote 2.6).

### Practice

In the pre-hospital environment, paramedics can identify atrial fibrillation (AF) and hypertension (HTN), but do not currently assess hyperlipidaemia in standard practice. There was a unanimous response for management of these patients (AF and HTN) to be onward referral to the patient’s GP, or transport to hospital for immediate management and stabilisation if indicated (Table 3, quotes 3.1 and 3.2).

**Table 3.** Participants’ comments surrounding ‘practice’ of paramedics.

Quotation reference	Quote
3.1	*‘Straight to the doctor, whether it’s a hospital doctor, or their own doctor, generally there’s someone far better placed to be able to deal with them than I am’* (participant 1)
3.2	*‘no one’s ever going to miss an undiagnosed AF, but it’s easier to miss somebody who’s a little bit portly’ … ‘you would never just walk out and not do something about that new AF’* (participant 1)
3.3	*‘we can give them the information for them to be able to take it forward, but what we probably could do with is a programme in which to facilitate that’* (participant 2)
3.4	*‘we’re probably better off referring them to more appropriate services, rather than full on trying to address the problem ourselves’ … ‘they have somebody who goes in, who’s a professional at that and engaging people, keeping them on track, and reducing those risk factors, then that might be more effective than my desperate plea at three o’clock in the morning’* (participant 1)

AF: atrial fibrillation.

Participants were keen to see an ‘*obesity or lifestyle referral pathway*’ formed, to assist their practice in this subject (Table 3, quote 3.3). It was felt that by having an integrated care network of other healthcare professionals that were more qualified to address these issues with patients, it would encourage them to use this practice more (Table 3, quote 3.4).

## Discussion

The findings demonstrated key themes of education and anxiety. These were discussed through how paramedics feel about their role, behaviour and practice in the identification of CVRFs.

The participants defined their role consistently to highlight their ability to educate patients, and this was in keeping with the Health and Care Professions Council (2014) standards of proficiency surrounding health promotion, as well as the College of Paramedics (2019) curriculum. This is encouraging; however, the group related the health promotion to the presenting complaint of the patient, when discussing their role in health promotion, rather than making full use of the ‘making every contact count’ initiative (Health Education England, 2021). This coincides with the participants’ suggestion of further training in the matter and can be viewed as a potential area for improvement to encourage paramedics to fulfil their role in this area.

Behaviours discussed by participants were largely surrounding barriers to their role, illustrating the fear and anxiety as one of the main themes. Due to lack of support from the ambulance service, alongside the fear of complaints, paramedics feel vulnerable when approaching a patient about their weight and other lifestyle choices, thus avoid contentious conversations. Lawrence et al. (2016) identified a gap in the research for further training schemes to be explored and recommended prioritising this area for development to enhance practitioners’ health promotion skills.

Another key area affecting the behaviour of paramedics when managing CVRFs was the fear of hypocrisy. Paramedics feel ill-equipped to maintain their own physical fitness. Hegg-Deloye et al. (2014) identified that obesity among paramedics is increasing, due to factors such as difficult and changeable shift patterns and lack of time for exercise. Hegg-Deloye et al. (2015) concerningly found many paramedics are at risk of developing CVD. Like many ambulance services in the United Kingdom, there is no minimum fitness level or fitness test required for ongoing employment.

Paramedics identified a lifestyle referral pathway, as part of an integrated care service, to be a key area for development. Reddy (2006) outlined guidance for obesity referral pathways in primary care, but there is no accessible alternative for an equivalent in the pre-hospital environment. Zaver (2020) also described exercise referrals that are being introduced to primary care. However, these are only accessible through a primary care practitioner, rather than directly from the pre-hospital environment.

### Limitations

The most significant limitation to this study was the sample size, and single focus group for data collection. Unfortunately, due to the time constraints of the study, it was not feasible to carry out multiple focus groups and meet data saturation. Data saturation was not met in any areas during this study. This is suggested to be the base of future research, in which to further explore the areas for development identified by this study.

The transferability of the findings is also limited due to the sample size and constriction of participants being from only one ambulance service. As the north east has relatively high levels of CVD in comparison to the UK average (Office for Health Improvement and Disparities, 2021), it would be beneficial to explore the views of paramedics from other UK ambulance services to improve the generalisation. The topic guide for the focus group is available upon request for researchers hoping to continue this exploration.

## Conclusion

Paramedics feel their contribution to the identification of CVRFs in the pre-hospital environment is through patient education. However, their behaviour and practice of this role is adversely affected by several barriers. To allow paramedics to fulfil their role in this area, the practicalities of how to encompass pre-hospital clinicians in wider health improvement need to be explored. Future research should seek to identify the practicalities for integrated care for health promotion, as well as service improvement for health and welfare of staff.

## Acknowledgements

We are grateful to the paramedics who took part in this study, and to the NHS host organisation, North East Ambulance Service NHS Foundation Trust, for their support and guidance.

## Author contributions

Both authors read and approved the final manuscript. JT conducted the study and subsequent analysis, with GM supervising. JT conducted the focus group, with GM to facilitate. Report written by JT and checked by GM. GM acts as the guarantor for this article.

## Conflict of interest

GM is the editor-in-chief of the *BPJ*.

## Ethics

Ethical approval for the study was not needed according to the HRA decision-making tool (Health Research Authority, 2020), as the participants were all NHS employees and the topic matter is non-sensitive. HRA approval was secured (298909, 5 May 2021), as was local NHS R&D (NEAS/2021/298909). Participants were provided with an information sheet alongside their consent form, to ensure consent was fully informed.

## Funding

The study received no direct funding. The authors completed the study as part of the NIHR Clinical Research Internship. The NIHR had no influence over the design, conduct, analysis or reporting of the study.
